# Melittin Exerts Beneficial Effects on Paraquat-Induced Lung Injuries in Mice by Modifying Oxidative Stress and Apoptosis

**DOI:** 10.3390/molecules24081498

**Published:** 2019-04-16

**Authors:** Bishoy El-Aarag, Mohamed Magdy, Mohamed F. AlAjmi, Shaden A.M. Khalifa, Hesham R. El-Seedi

**Affiliations:** 1 Biochemistry Division, Chemistry Department, Faculty of Science, Menoufia University, Shebin El-Koom 32512, Egypt; 2 Division of Chemistry and Biotechnology, Graduate School of Natural Science and Technology, Okayama University, Okayama 7008530, Japan; 3 Department of Chemistry, Faculty of Science, Menoufia University, Shebin El-Koom 32512, Egypt; mohamed_magdy201237@yahoo.com (M.M.); hesham.el-seedi@ilk.uu.se (H.R.E.-S.); 4 Department of Pharmacognosy, College of Pharmacy, King Saud University, Riyadh 11451, Saudi Arabia; malajmii@ksu.edu.sa; 5 Department of Molecular Biosciences, The Wenner-Gren Institute, Stockholm University, S-106 91 Stockholm, Sweden; shaden.khalifa@su.se; 6 Division of Pharmacognosy, Department of Medicinal Chemistry, Uppsala University, Biomedical Centre, Box 574, SE-75123 Uppsala, Sweden; 7 International Center for Chemical and Biological Sciences, University of Karachi, Karachi-75270, Pakistan

**Keywords:** paraquat, lung injury, melittin, oxidative stress, apoptosis

## Abstract

Melittin (MEL) is a 26-amino acid peptide with numerous biological activities. Paraquat (PQ) is one of the most widely used herbicides, although it is extremely toxic to humans. To date, PQ poisoning has no effective treatment, and therefore the current study aimed to assess for the first time the possible effects of MEL on PQ-induced lung injuries in mice. Mice received a single intraperitoneal (IP) injection of PQ (30 mg/kg), followed by IP treatment with MEL (0.1 and 0.5 mg/kg) twice per week for four consecutive weeks. Histological alterations, oxidative stress, and apoptosis in the lungs were studied. Hematoxylin and eosin (H&E) staining indicated that MEL markedly reduced lung injuries induced by PQ. Furthermore, treatment with MEL increased superoxide dismutase (SOD), catalase (CAT), and glutathione peroxidase (GPx) activity, and decreased malonaldehyde (MDA) and nitric oxide (NO) levels in lung tissue homogenates. Moreover, immunohistochemical staining showed that B-cell lymphoma-2 (Bcl-2) and survivin expressions were upregulated after MEL treatment, while Ki-67 expression was downregulated. The high dose of MEL was more effective than the low dose in all experiments. In summary, MEL efficiently reduced PQ-induced lung injuries in mice. Specific pharmacological examinations are required to determine the effectiveness of MEL in cases of human PQ poisoning.

## 1. Introduction

Paraquat (PQ, 1,1-dimethyl-4,4-bipyridinium dichloride) is one of the most widely used herbicides globally, mainly in developing countries [1]. PQ is highly toxic to humans and is a primary cause of death [2,3]. Therefore, PQ poisoning is an important public health problem [4,5]. PQ induces pulmonary fibrosis, and oxidative stress plays an important role in this process since the oxidation–reduction reactions of PQ produce reactive oxygen species (ROS). These species damage alveolar epithelial cells and are assumed to be responsible for direct cell injury [6,7,8].

PQ causes lung toxicity and fibrosis through several molecular mechanisms, such as caspase cleavage, matrix metalloproteinase-9 (MMP-9), peroxisome proliferator-activated receptor-gamma (PPAR-γ), nuclear factor-kappaB (NF-κB), Jun N-terminal kinase/p38 mitogen-activated protein kinase (JNK/p38 MAPK), Nuclear factor-like2/NADPH oxidase4 (Nrf2/Nox4) redox balance, and transforming growth factor-beta/small mothers against decapentaplegic3 (TGF-β1/Smad3) signaling pathways [9,10,11,12,13,14].

Bee venom (BV), produced by honey bees (Apis mellifera L.), is a mixture of several biologically active peptides and toxins, including melittin (MEL), enzymes, and bioactive amines with pharmaceutical properties [15]. Several beneficial roles of BV were reported, for instance radioprotective [16], anti-mutagenic [17], anti-nociceptive [18,19], anti-cancer [20,21,22,23], and anti-inflammatory [24,25,26] activities.

MEL, a 26-amino acid peptide, is the main active pharmacological constituent of BV. This peptide comprises approximately 50% of the dry weight of bee venom [27]. MEL is a water-soluble peptide, weighing 2840 Da, and with a chemical formula C_131_H_229_N_39_O_31_. Also, it is characterized as a linear, cationic, hemolytic, and amphipathic peptide [28]. MEL has numerous biological activities, including anti-viral, anti-bacterial, anti-fungal, anti-parasitic, and anti-tumor. These activities were claimed to the non-selective cytolytic peptide that disrupts all prokaryotic and eukaryotic cell membranes [29,30,31,32]. The cytolytic mechanism of MEL relies on its ability to bind with negatively charged membrane surfaces, leading to disturbing the integrity of phospholipid bilayers through pore formation that improves their permeability and eventually leads to cell lysis [33]. Since the membrane potential of the tumor cells is higher and tumor cells are less likely to develop resistance to a membrane pore formation, MEL was considered an attractive candidate for cancer chemotherapy, causing more damage to the tumor cell membranes [34,35,36,37].

Both apoptosis and cell proliferation are known to be important pathophysiological processes in acute lung injury, especially for the cytoprotection and regeneration of lung epithelial cells after injury [38,39]. B-cell lymphoma-2 (Bcl-2) is an anti-apoptotic molecule that prevents oxidative stress-related damage and cell death [40]. Survivin is a protein that inhibits apoptosis [41] and regulates cell division, and is therefore a key mediator of cell protection in acute lung injuries [42]. Ki-67 is a nuclear protein that serves as a marker of proliferation, and its expression is a reliable marker of tissue fibrotic transformation [43]. Oxidative stress plays an important role in lung fibrosis, as elevated levels of ROS are accompanied by reduced antioxidant enzyme levels [44,45,46]. Additionally, antioxidants are effective tools for reducing lung injury in experimental animals [47,48]. Therefore, the present study aimed to explore the possible antioxidant and apoptotic activities of MEL against PQ-induced lung injuries in mice.

## 2. Results

### 2.1. Histopathological Analysis of the Effects of MEL on Lung Tissues

**Figure 1:**
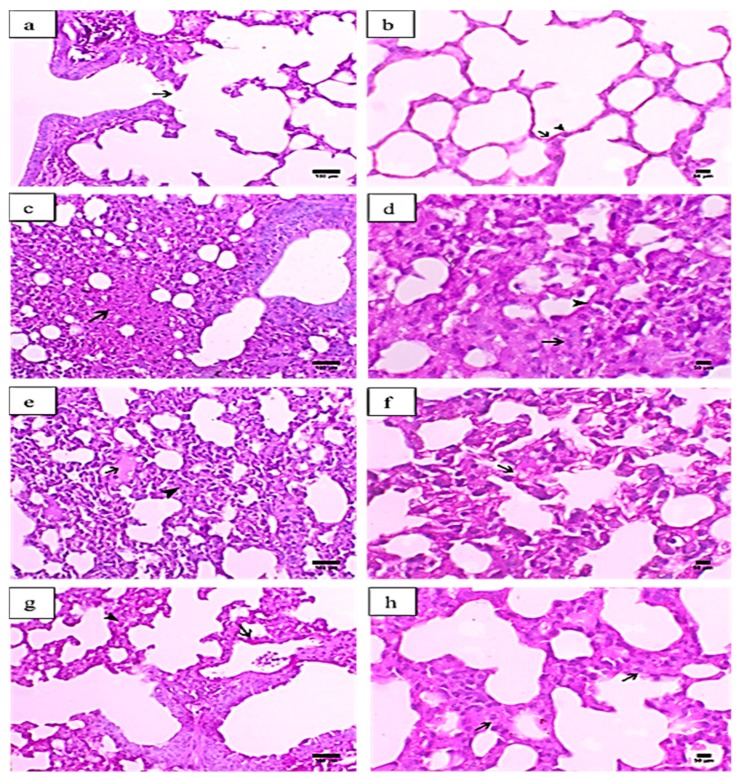
Histopathological graphs of lung sections from all groups. The lung sections were analyzed by hematoxylin and eosin (H&E) staining. Control group (**a**,**b**): (**a**) indicated normal terminal bronchiole ended with respiratory portion (arrow) (X200, scale bar = 100 µm), (**b**) showed alveoli lined with alveolar epithelial cells pnemocyte type I (arrow) and type II (arrowhead) (X400, scale bar = 50 µm). Paraquat (PQ) group (**c**,**d**): (**c**) presented necrosis in the alveolar tissue (arrow) (X200, scale bar = 100 µm), (**d**) showed excessive interstitial tissue thickening associated with septal cell proliferation (arrow) and alveolar hyaline membrane formation (arrowhead) (X400, scale bar = 50 µm). PQ + MEL (melittin) (0.1 mg/kg) group (**e**,**f**): (**e**) illustrated mild focal serous exudate within the alveoli (arrow) accompanied with a moderate degree of inter-alveolar interstitial tissue thickening (arrowhead) (X200, scale bar = 100 µm) (**f**) presented a moderate degree of thickening in the inter-alveolar septa and marked hyperplasia of pneumocyte type II in the lining epithelium (arrow) (X400, scale bar = 50 µm). PQ + MEL (0.5 mg/kg) group (**g**,**h**): (**g**) displayed decrease in the inter-alveolar thickening (arrowhead) with marked hyperplasia of pneumocyte type II (arrow) (X200, scale bar = 100 µm) (**h**) presented a decrease in the inter-alveolar septa (arrowhead) with an increase in the alveolar spaces associated with hyperplasia of pneumocyte type II (arrow) (X400, scale bar = 50 µm).

Figure 1a,b presents the histopathological examination of the lung tissues of normal control mice, showing normal bronchial and alveolar tissues. The bronchi exhibited a normal cuboidal epithelium that ended with a terminal bronchus that communicated directly with the respiratory portion. The respiratory portion consisted of many alveoli that were lined with two types of alveolar epithelial cells, type I pneumocytes (flattened epithelial cells), and type II pneumocytes (mostly cuboidal cells). The lung tissues of mice injected with PQ showed necrosis of the epithelium lining the bronchus, associated with hyaline membrane formation. The lesions were mostly peribronchial in nature. Moreover, the peribronchial areas revealed marked lymphoid hyperplasia, with the collapse and atelectasis of the neighbouring peribronchial alveoli. The alveolar space was decreased due to marked alveolar tissue necrosis, mixed cellular infiltration, and vascular exudate. The interstitial tissue revealed marked thickening, associated with septal cell proliferation, as well as mononuclear inflammatory cell infiltration. The alveolar lumen was also decreased due to the formation of eosinophilic hyaline membranes, as shown in Figure 1c,d.

The lung tissues of mice injected with PQ and treated with MEL (0.1 mg/kg) demonstrated a mild to moderate increase in the alveolar space in association with a decrease in interstitial tissue thickening. The alveolar wall was markedly corrugated and usually accompanied by a marked proliferation of type II pneumocytes, which usually showed a vacuolated cytoplasm consistent with crystalline bodies (Figure 1e,f). Additionally, mice treated with MEL at a dose of 0.5 mg/kg exhibited an obvious decrease in interstitial tissue thickening, associated with decreases in both collagen deposition and septal cell proliferation. The type II alveolar cells showed hypertrophy, with increased vacuolation within the cytoplasm. Peribronchial infiltration was markedly decreased, as indicated in Figure 1g,h.

### 2.2. Effect of MEL on Tissue Malonaldehyde (MDA) Levels

**Figure 2:**
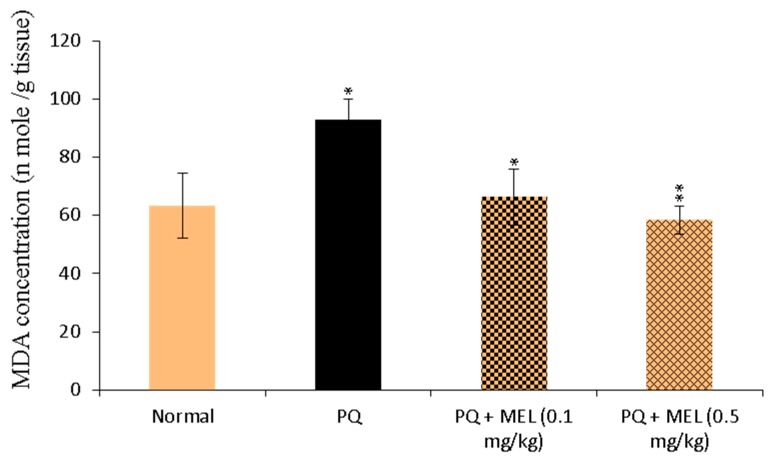
Effect of treatment with melittin (MEL) on the levels of malonaldehyde (MDA) in lung tissue homogenate. MDA (lipid peroxidation index) levels were determined in the lung tissue homogenate using MDA colorimetric assay. The absorbance of the formed product was measured at 534 nm. The results were expressed as nmol MDA per g tissue. Data are presented as mean ±SD. Significantly (* *p* < 0.05, ** *p* < 0.01) different from PQ group.

The levels of MDA in the lung tissue homogenate were measured as an indicator of lipid peroxidation. As shown in Figure 2, lung tissue homogenate MDA levels were significantly (*p* < 0.01) increased in PQ-treated mice compared to those in the control group. In contrast, mice treated with MEL, especially the high dose, exhibited a significant (*p* > 0.01) reduction in PQ-induced MDA production compared to that in the PQ group.

### 2.3. Effect of MEL on Tissue Nitric Oxide (NO) Levels

**Figure 3:**
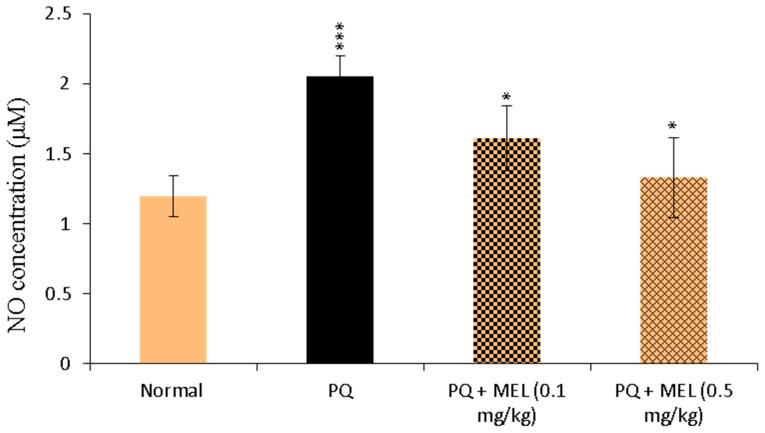
Effect of treatment with melittin (MEL) on the levels of nitric oxide (NO) level in lung tissue homogenate. The level of NO in the lung homogenate was measured according to the Griess method, using NO colorimetric assay. The formed azo dye was measured at 540 nm. NO levels were measured and expressed as µM. Data are presented as mean ±SD. Significantly (* *p* < 0.05, *** *p* < 0.001) different from PQ group.

As shown in Figure 3, NO levels in the lung tissue homogenates were significantly (*p* < 0.01) elevated in the PQ-induced lung fibrosis group. On the other hand, the administration of MEL significantly (*p* < 0.05) reduced the level of NO compared to that in the PQ group, with levels close to those in the control group (Figure 3).

### 2.4. Effect of MEL on Tissue Superoxide Dismutase (SOD) Activity

**Figure 4:**
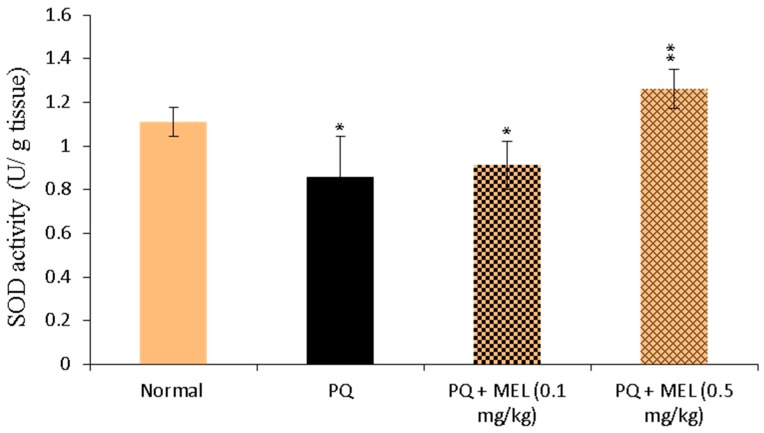
Effect of treatment with melittin (MEL) on superoxide dismutase (SOD) activity in lung tissue homogenate. SOD activity was measured according to Beyer method, using SOD colorimetric assay. SOD activity was measured and expressed as U/g tissue. Data are presented as mean ±SD. Significantly (* *p* < 0.05, ** *p* < 0.01) different from PQ group.

Compared to the control, the administration of PQ significantly (*p* < 0.01) diminished the level of SOD in the lung tissue homogenate, as shown in Figure 4. Treatment with MEL, especially the high dose, significantly (*p* < 0.01) increased the SOD activity compared to that in the PQ group.

### 2.5. Effect of MEL on Tissue Catalase (CAT) Activity

**Figure 5:**
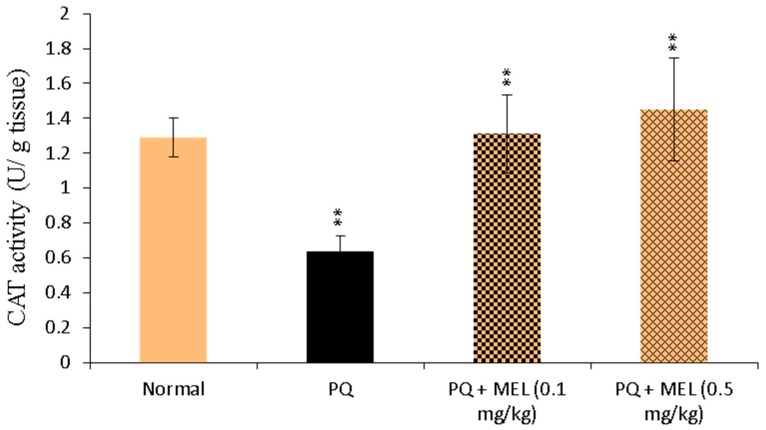
Effect of treatment with melittin (MEL) on catalase (CAT) activity in lung tissue homogenate. CAT was determined in the lung tissue homogenate through the Aebi method, using CAT colorimetric assay. The color intensity was measured, and CAT activity was determined and expressed as U/g tissue. Data are presented as mean ±SD. Results are significantly (** *p* < 0.01) different from the PQ group.

As presented in Figure 5, the activity of CAT in the lung tissue homogenate was significantly reduced in the PQ group compared to that in the control group. Compared to PQ alone, MEL treatment induced a significant increase in CAT activity, especially in the PQ + MEL (0.5 mg/kg) group.

### 2.6. Effect of MEL on Tissue Glutathione Peroxidase (GPx) Activity

**Figure 6:**
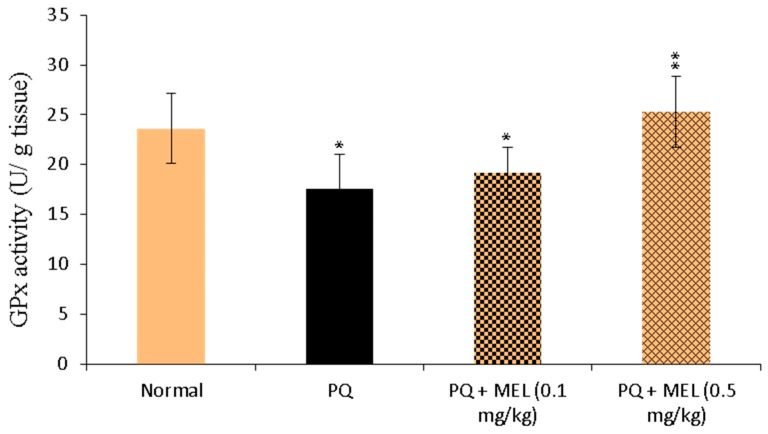
Effect of treatment with melittin (MEL) on glutathione Peroxidase (GPx) activity in lung tissue homogenate. GPx was measured in the lung tissue homogenate by UV spectroscopic method. The decrease in the absorbance was measured at 340 nm. The level of GPx was measured and expressed as U/g tissue. Data are presented as mean ±SD. Results are significantly (* *p* < 0.05, ** *p* < 0.01) different from PQ group.

The measurement of lung antioxidant GPx levels showed that PQ reduced GPx levels, as indicated in Figure 6. However, the administration of MEL significantly increased the GPx level in the lung homogenate compared to that in the PQ group.

### 2.7. Effect of MEL on Bcl-2 Levels in the Lung Tissue

**Figure 7:**
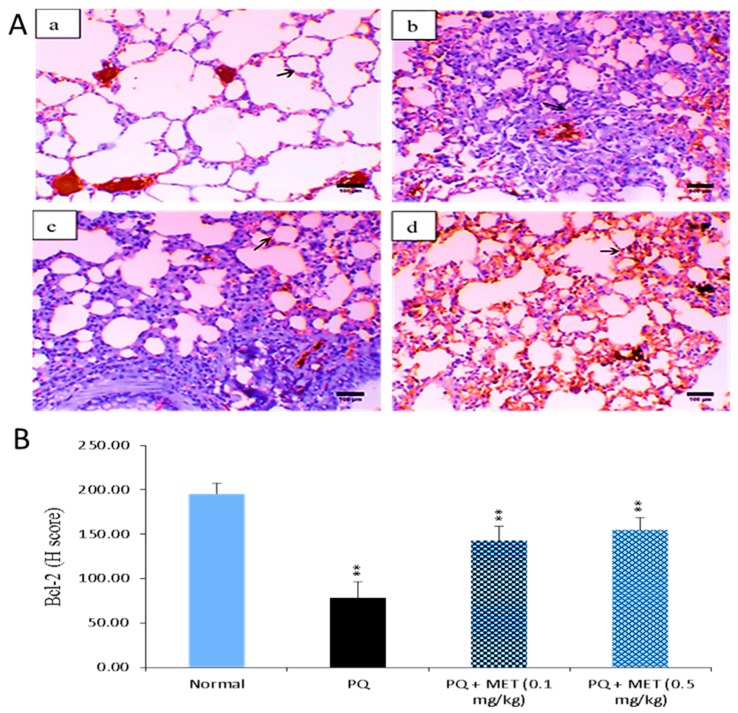
Effect of treatment with melittin (MEL) on the B-cell lymphoma-2 (Bcl-2) protein level in lung tissues. **A**: Representative lung immunohistochemical graphs (a: control group, b: paraquat (PQ) group, c: PQ + MEL (0.1 mg/kg) group, d: PQ + MEL (0.5 mg/kg) group); (**a**) showed a noticeable expression of bcl-2 immunostaining in the alveolar lining epithelium (arrow). (**b**) presented marked decrease in bcl-2 expression within the alveolar lining epithelium (arrow). (**c**) displayed mild to moderate increase of bcl-2 expression in the alveolar epithelium (arrow). (**d**) illustrated an obvious increase in bcl-2 expression within the alveolar epithelium (arrow). Magnification = X200 and scale bar = 100 µm. **B**: Statistical analysis of Bcl-2 H-score. Data are expressed as mean ±SD. Significantly (** *p* < 0.01) different to the PQ group.

As displayed in Figure 7, the administration of PQ caused a significant (*p* < 0.01) reduction in Bcl-2 levels compared to those in the control group. Treatment with MEL restored the protein levels of Bcl-2 compared to those of the normal control. In particular, the high dose of MEL (0.5 mg/kg) significantly (*p* < 0.01) elevated the Bcl-2 level to near normal.

### 2.8. Effect of MEL on Survivin Levels in the Lung Tissue

**Figure 8:**
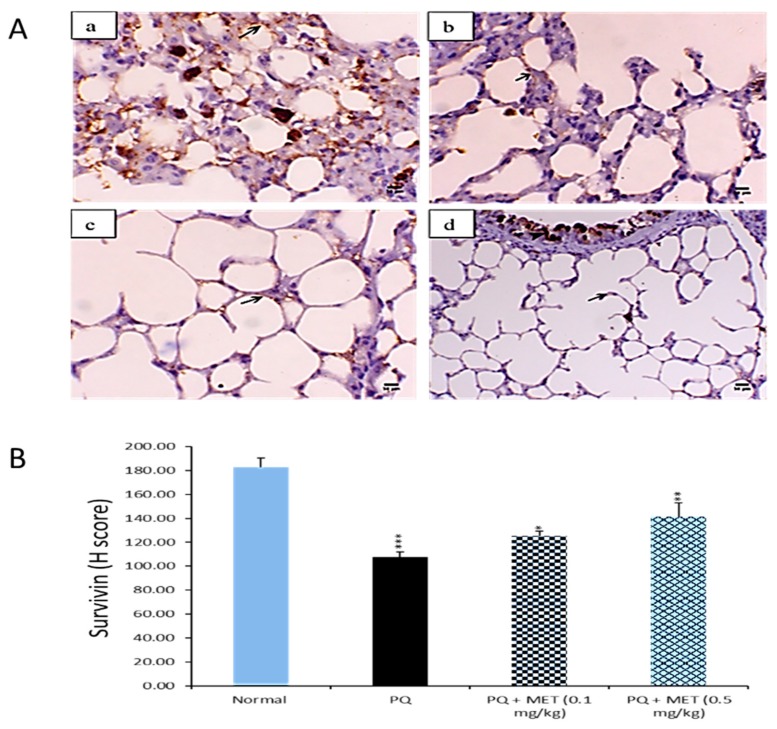
Effect of treatment with melittin (MEL) on the survivin protein level in lung tissues. **A**: Representative lung immunohistochemical graphs (a: control group, b: paraquat (PQ) group, c: PQ + MEL (0.1 mg/kg) group, d: PQ + MEL (0.5 mg/kg) group); (**a**) showed a strong survivin expression within the alveolar epithelium (arrow) (X400, scale bar = 50 µm). (**b**) presented a low to mild survivin expression within the alveolar epithelium (arrow) (X400, scale bar = 50 µm). (**c**) indicated a moderate survivin expression in the alveolar epithelium (arrow) as well as in the bronchial epithelium (arrowhead) (X400, scale bar = 50 µm). (**d**) illustrated an increase in the survivin expression in the alveolar epithelium (arrow) and in the bronchial epithelium (arrowhead) (X200, scale bar = 100 µm). **B**: Statistical analysis of survivin H-score. Data are expressed as mean ±SD. Significantly (* *p* < 0.05, ** *p* < 0.01, *** *p* < 0.001) different from PQ group.

Survivin is a protein that inhibits apoptosis and promotes cell proliferation [49]. Compared to levels in the control group, the survivin level was significantly (*p* < 0.001) declined in injured lung tissues after PQ injection. In contrast, survivin levels elevated in mice injected with PQ followed by MEL treatment. A high dose of MEL significantly (*p* < 0.01) augmented survivin levels, with an effect stronger than that observed in the other groups (Figure 8).

### 2.9. Effect of MEL on Ki-67 Levels in the Lung Tissue

**Figure 9:**
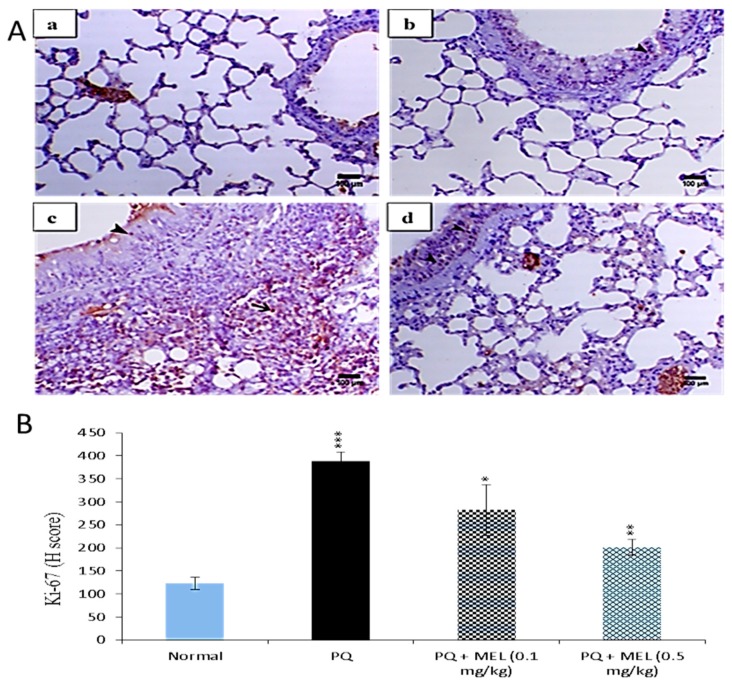
Effect of treatment with melittin (MEL) on Ki-67 protein level in lung tissues. **A**: Representative lung immunohistochemical graphs (a: control group, b: paraquat (PQ) group, c: PQ + MEL (0.1 mg/kg) group, d: PQ + MEL (0.5 mg/kg) group); (**a**) showed mild expression of Ki-67 immunostaining within the alveolar and bronchial lining epithelium (arrow). (**b**) presented an obvious increase in the inter-alveolar septa (arrow) as well as in the necrotic epithelial bronchial lining (arrowhead). (**c**) illustrated a marked decrease in Ki-67 expression within the alveolar and bronchial epithelium (arrowhead). (**d**) indicated a noticeable decline in Ki-67 expression within the alveolar and bronchial epithelium (arrowhead). Magnification = X200 and scale bar = 100 µm. **B**: Statistical analysis of Ki-67 H-score. Data are expressed as mean ±SD. Significantly (* *p* < 0.05, ** *p* < 0.01, *** *p* < 0.001) different from the PQ group.

Lung fibrosis is characterized by fibroblast proliferation and extracellular matrix remodeling, which leads to respiratory failure [50]. Figure 9 reveals that PQ significantly (*p* < 0.01) elevated the expression level of Ki-67 in lung tissues compared to those in the control group. Treatment with either a low or high dose of MEL can decrease these elevated Ki-67 levels. MEL (0.5 mg/kg) significantly (*p* < 0.01) decreased the expression of Ki-67, with an effect stronger than that observed in the other groups.

## 3. Discussion

PQ intoxication is an important health problem because it causes global environmental intoxication with severe toxic effects, including pulmonary fibrosis [51]. The toxic effects of PQ result from its ability to produce ROS through redox cycling processes, which leads to mitochondrial oxidative stress and potential cell death [52,53,54,55]. The induction of oxidative stress and apoptosis may be involved in the experimental lung damage induced by PQ. Therefore, it was important to examine whether MEL would be effective against PQ-induced lung damage in mice. Thus, the current study aimed to investigate the apoptotic and antioxidant activity of MEL in a mouse model of PQ-induced lung fibrosis.

A histological evaluation of hematoxylin and eosin (H&E) stained lung sections from the PQ group showed necrosis of the bronchial epithelium, lymphoid hyperplasia with a marked collapse and atelectasis of the neighbouring peribronchial alveoli, a marked decrease in alveolar space, thickening interstitial tissue associated with septal cell proliferation, and mononuclear inflammatory cell infiltration. Our results were comparable to previously reported histopathological examinations of lung fibrosis induced by PQ in mice [56,57,58,59]. Treatment with MEL (0.1 gm/kg) twice per week for four consecutive weeks could improve the histopathological changes in the lung tissues, and the antifibrotic effects were more obvious in the lung tissues of mice treated with MEL (0.5 gm/kg) than in the PQ group, suggesting that treatment with a high dose of MEL may have a more potent effect for restoring the histopathological changes induced by PQ.

Lipid peroxidation is associated with several harmful effects, such as increased membrane rigidity and reduced mitochondrial survival [60]. One of the most commonly used indicators of lipid peroxidation is MDA, the product of lipid peroxidation. Our study revealed that MDA levels in the lung tissue homogenate were significantly increased in PQ-injected mice compared to those in the control group. These results are consistent with previously reported studies [61,62]. PQ induces the accumulation of free radicals, which leads to lipid peroxidation and the overproduction of oxidation markers such as MDA [63,64]. Our results demonstrated that compared to the control, MEL significantly reduced this elevated MDA production. MDA can indirectly reflect the degree to which ROS attack cells in the lung tissue and the antioxidant ability of the body. Therefore, we suggest that MEL decreased lipid peroxidation through its ability to effectively reduce MDA levels.

NO is a reactive nitrogen species (RNS) and mediates nitrative stress via the nitration of proteins, lipids, and nucleic acids [45,65]. It has been reported that RNS are involved in PQ-mediated lung injuries, accompanied by augmented NO production, in experimental animals injected with PQ [66,67,68,69,70]. These findings are consistent with the results of our study, which showed that PQ significantly increased NO production, which highlighted the pathophysiological importance of NO in PQ-mediated lung injuries. In contrast, treatment with MEL significantly reduced NO production in PQ-exposed mice, which explains the ability of MEL to reverse the effects of PQ-induced NO production in lung tissues. The lung contains a group of antioxidant enzymes, including SOD, CAT, and GPx.

The activities of these enzymes reflect the tissue oxidant–antioxidant balance [71]. SOD catalyzes the dismutation of superoxide anions into oxygen and hydrogen peroxide, and plays a vital role in antioxidant defense against superoxide radicals in cells [72]. CAT catalyzes the decomposition of hydrogen peroxide into water and oxygen. Additionally, it deactivates the harmful effects of peroxide radicals in biological systems [73]. GPx is an effective scavenger of cellular peroxides, and this process is catalyzed by the oxidation of glutathione to glutathione disulfide [74]. A decline in SOD, CAT, and GPx levels during PQ-induced lung injury and an increase during treatment were previously reported [3,72,75]. These reports are consistent with the results of the current study, which revealed a reduction in the activity of antioxidant enzymes in the PQ group. The reduced levels of these enzymes may relate to enzyme consumption due to the neutralization of excess superoxide anions and hydrogen peroxide in the lung tissues. On the other hand, treatment with MEL significantly increased the levels of antioxidant enzymes compared to those in the PQ group, highlighting the antioxidant effects of MEL in neutralizing the oxidant activity of PQ-induced free radicals in the lung tissue.

Apoptosis plays a crucial role in wound repair and in lung epithelial injuries that lead to fibrosis. Markedly increased alveolar epithelial cell apoptosis has been reported in lung fibrosis [76,77]. Similarly, previous studies showed the role of decreased Bcl-2 expression in increasing epithelial cell death and the role of increased Bcl-2 expression in resistance to apoptosis [78]. Our results showed that the administration of PQ caused a significant reduction in Bcl-2 protein levels and that treatment with MEL increased these reduced Bcl-2 levels. Our findings were consistent with other studies, showing the ability of high levels of the anti-apoptotic protein Bcl-2 to prevent apoptosis in lung fibroblasts [79,80,81].

To gain another insight into the anti-apoptotic activity of MEL, the survivin expression of each treated group was measured. Our results revealed that survivin protein levels were significantly decreased in the PQ-injured lung tissues of mice. Conversely, the treatment with MEL increased survivin protein levels, indicating that MEL can reverse the fibrosis induced by PQ. It has been reported that survivin expression is increased in idiopathic pulmonary fibrosis fibroblasts and that it enhanced the susceptibility of these cells to apoptosis by inhibiting survivin [82]. Also, survivin mediates cytoprotection in acute lung injuries by a mechanism that depends on the inhibition of apoptosis [41]. Furthermore, survivin overexpression can protect gastric epithelial cells from ethanol-induced apoptosis [83].

Ki-67, a nuclear antigen present in proliferating cells, is the most widely used proliferation-associated marker [84]. The expression of Ki-67 is detected in lung epithelial cells [85,86]. Our immunohistochemical results for Ki-67 showed that the level of Ki-67 was significantly increased in the lung tissues of mice administered with PQ. This finding is consistent with the previously reported findings that the expression of Ki-67 was significantly elevated in idiopathic pulmonary fibrosis [87]. Additionally, it has been reported that the administration of bleomycin, an inducer of lung fibrosis, increased the level of Ki-67 in lung tissues [88]. On the other hand, treatment with MEL can markedly reduce this elevated level of proliferation to a level near that of the control group. This finding revealed the potent ability of MEL to inhibit the increase in the Ki-67 proliferation marker and consequently reduce cell proliferation in lung fibrosis induced by PQ.

The methods of PQ and MEL injection are the limitations of the present study, where it is more convenient to induce lung injuries through oral intake or inhalation of PQ. Additionally, MEL is a large size peptide (26 amino acid); therefore, there is a probability that it can be digested in the gastrointestinal tract. Thus, the effects of MEL through intravenous (IV) or subcutaneous (SC) injection are more appreciated.

## 4. Materials and Methods

### 4.1. PQ and MEL

PQ was purchased from Sigma (St. Louis, MO, USA) and dissolved in saline solution (0.9% NaCl) to prepare the working concentration used in the present study.

### 4.2. MEL Isolation, Purification, and Identification

MEL was isolated from bee venom collected from apiary hybrid Carniolan honeybees according to Benton’s method [89]. The isolation process was carried out using Äkta HPLC, a Phenomenex C18 column (250 × 10 mm i.d., 5 µm, 300 Å), with a linear gradient from 0 to 100% buffer B (60% (*v*/*v*) acetonitrile (can), 0.1% (*v*/*v*) trifluoroacetic acid (TFA)) over 60 min with a flow rate of 4 mL/min, then the peptide peak was manually collected. The peptide was purified by a Phenomenex C18 column (250 × 4.6 mm i.d., 5 µm, 300 Å), run with a linear gradient from 0 to 100% buffer B over 60 min at a flow rate of 1 mL/min. MEL was identified using liquid chromatography-mass spectrometry (LC-MS) (Thermo Finnigan, San Jose, CA, USA) and tandem mass spectrometry (MS-MS) fragmentation (Waters, Milford, MA, USA) [90,91].

### 4.3. Mice

A total of 40 adult male Swiss albino mice (8 weeks old) with an average body weight of 30 g were used in the current study. All animal experiments were performed according to animal ethics rules and regulations, approved by the Ethical Committee for Laboratory Animals of Science Faculty Menoufia University (No.: ECLA-SFMU-15116). The animals were maintained in standard cages under controlled temperature conditions with a 12 h light/dark cycle and given food and water ad libitum.

### 4.4. Experimental Design

Lung fibrosis was induced in male Swiss albino mice by intraperitoneal (IP) administration of PQ (30 mg/kg) [92]. All groups except group 1 were injected intraperitoneally with a single dose of PQ; group 1 was included as the normal control group. Two hours after the induction of fibrosis, the mice were divided into three groups (10 mice each) as follows: group 2 (PQ group), the mice were intoxicated with PQ (30 mg/kg); group 3 (PQ + MEL (0.1 mg/kg)), the mice were injected with PQ followed by the IP administration of MEL (0.1 mg/kg); and group 4 (PQ + MEL (0.5 mg/kg)), the mice were injected with PQ followed by the IP administration of MEL (0.5 mg/kg). MEL treatment was started 2 h after the PQ injection twice per week for four consecutive weeks. The two doses (0.1 and 0.5 mg/kg) were selected according to a lethal dose study (data not shown) and represent 1/25 and 1/5 of the LD_50_, respectively.

### 4.5. Preparation of Lung Tissue Homogenate

After the treatment period (four consecutive weeks), mice were anaesthetized with diethyl ether and sacrificed. Lung tissues were removed, and the left lobe of the lung was immediately fixed in 10% neutral buffered formalin for histological and immunohistochemical examinations. The right lobe of the lung was used to prepare a tissue homogenate in a potassium phosphate buffer, pH 7.4. The obtained homogenates were aliquoted and immediately frozen at −80 °C for antioxidant enzyme detection.

### 4.6. Histological Examination

The histological examination was performed as previously reported [93]. Briefly, the left lobe of the lung was fixed in 10% formaldehyde. After fixation, the lung tissues were embedded in paraffin wax and cut into 5 μm thick sections, followed by staining with H&E. Finally, the slides were examined under a light microscope.

### 4.7. Determination of Tissue MDA Levels

The lipid peroxidation level was measured according to a previously reported method [94]. MDA levels are an index of lipid peroxidation and were determined in the lung tissue homogenate using an MDA assay kit. Briefly, thiobarbituric acid reacts with MDA in the homogenate to form a thiobarbituric acid reactive product. The absorbance of the formed product was measured at 534 nm. The results were expressed as nmol MDA per g tissue.

### 4.8. Determination of Tissue NO Levels

The level of NO in the lung homogenate was measured according to the Griess method [95], using an NO assay kit. Briefly, in an acidic medium, nitrite is converted to nitrous acid, and the formed acid then reacts with sulphanilamide. The formed diazonium is coupled with *N*-(1-naphthyl) ethylenediamine to form azo dye. The colored dye was measured at 540 nm. NO levels were measured and expressed as µM.

### 4.9. Determination of Tissue SOD Activity

SOD is a metalloenzyme that catalyzes the dismutation of the superoxide anion to molecular oxygen and hydrogen peroxide. SOD activity was measured according to the Beyer method [96], using a SOD assay kit. SOD activity was measured and expressed as U/g tissue.

### 4.10. Determination of Tissue CAT Activity

CAT was determined in the lung tissue homogenate through the method of Aebi [97], using a CAT assay kit. Briefly, CAT reacted with a known quantity of H_2_O_2_. The reaction was stopped with a catalase inhibitor after exactly one minute. In the presence of peroxidase, the remaining H_2_O_2_ reacts with 3,5-dichloro-2-hydroxybenzene sulfonic acid and 4-aminophenazone to form a chromophore with a color intensity that is inversely proportional to the amount of catalase in the original sample. CAT activity was measured and expressed as U/g tissue.

### 4.11. Determination of Tissue GPx Activity

GPx catalyzes the thiol-dependent reduction of H_2_O_2_ and organic hydroperoxide, and therefore plays a protective role under oxidative stress conditions. GPx was measured in the lung tissue homogenate according to the reported method [98], using a GPx assay kit. The assay was based on the oxidation of NADPH to NADP^+^, which is accompanied by a decrease in the absorbance at 340 nm. The results were expressed as U/g tissue.

### 4.12. Immunohistochemical Detection of Bcl-2, Survivin, and Ki-67

Paraffin sections with a thickness of 5 μm were dewaxed in xylene and rehydrated in graded alcohol, followed by antigen retrieval using microwave heating (20 min; 10 mmol/citrate buffer, pH 6.0). The sections were immersed in 0.3% H_2_O_2_ for 30 min to block endogenous peroxidase activity, followed by blocking with 5% non-fat dry milk for 30 min. The sections were then incubated with primary monoclonal mouse anti-Bcl-2 (1:60 (v/v)) (BioGenex, Fremont, CA, USA), anti-survivin, and anti-Ki-67 antibodies (1:100 (v/v)) (Lab Vision, Thermo Fischer Scientific, Glasgow, UK) overnight at room temperature (RT). After washing three times with PBS (pH 7.4) for 5 min each, the sections were incubated with a secondary horseradish peroxidase (HRP)-conjugated anti-mouse antibody for 30 min at RT and then developed by diaminobenzidine (DAB). The sections were stained with haematoxylin for 5 min. The H-scores of Bcl-2, survivin, and Ki-67 were evaluated using a light microscope.

### 4.13. Statistical Analysis

The results were expressed as the mean ± standard deviation (SD). The statistical significance was assessed by the Student’s t-test for comparisons of two groups, and one-way analysis of variance (ANOVA) followed by Tukey’s post-hoc test for multiple group comparisons, using GraphPad Prism 6 (GraphPad Software Inc., San Diego, CA, USA). A *p* value of < 0.05 was considered statistically significant.

## 5. Conclusions

Our study showed for the first time that MEL could be a helpful agent for the treatment of lung injuries induced by PQ. MEL exerted potentially protective antioxidant effects by increasing SOD, GPx, and CAT activities and decreasing lipid peroxidation and NO levels. MEL also exhibited anti-apoptotic effects by increasing Bcl-2 and survivin expressions. Moreover, it declined the expression level of Ki-67. Additional pharmacological examinations are required to determine the effectiveness of MEL in human PQ poisoning.
